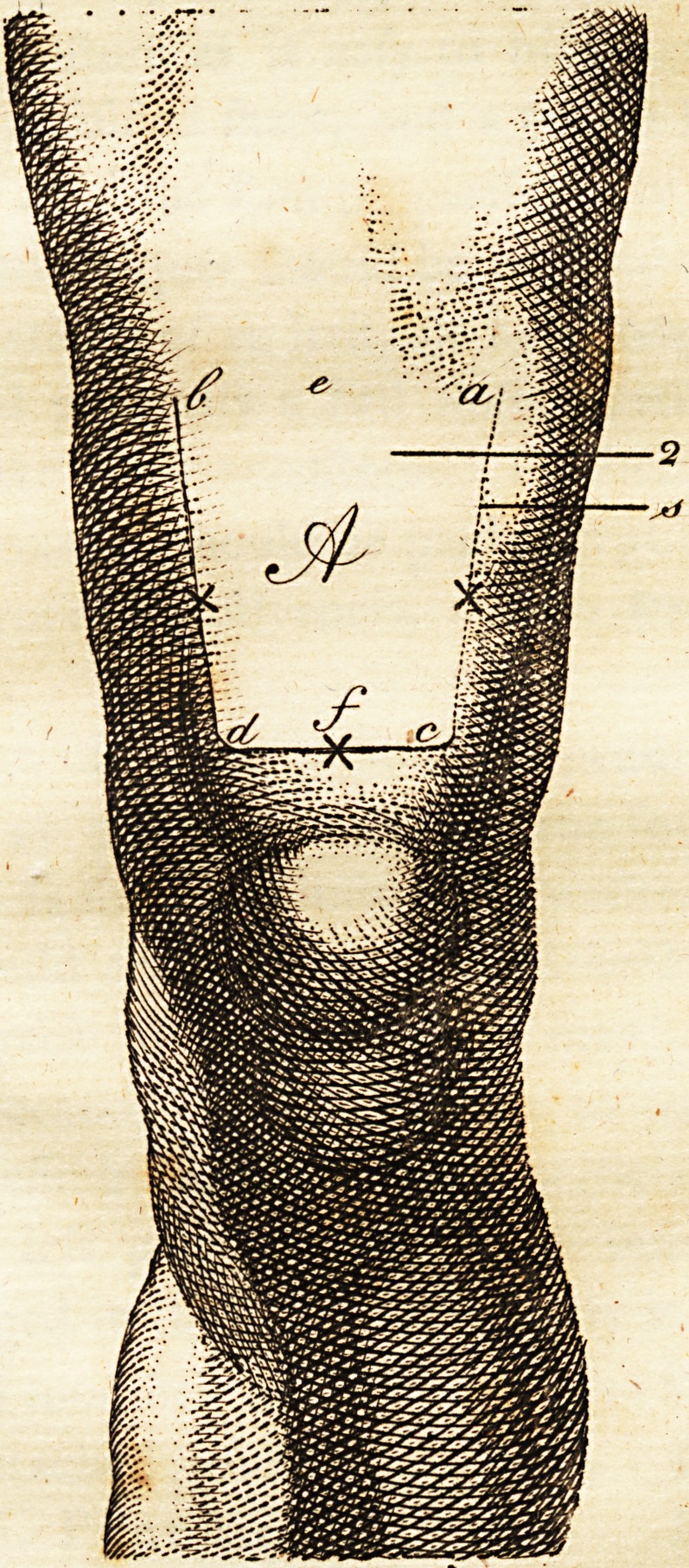# Practical Observations on Amputation

**Published:** 1786

**Authors:** James Lucas

**Affiliations:** one of the Surgeons of the General Infirmary at Leeds


### LONDON MEDICAL JOURNAL,

FOR THE YEAR 1786,

PART THE THIRD.

### I. *Practical Observations on Amputation. Communicated in a Letter to Dr.* Simmons, *F. R. S. by Mr.* James Lucas, *one of the Surgeons of the General Infirmary at Leeds.*

IT often requires as much diſcernment to determine when amputation may with ſafety be poſtponed, as to ſay when it is abſolutely neceſſary.

In caſes where the life of the patient is not immediately concerned, every remedy, which the ſkill of the ſurgeon can ſuggeſt, ſhould be preferred to operation. In caſes of accident, it is frequently neceſſary to decide within a few hours, or no ſafe opportunity for the operation may afterwards offer. Endeavours to heal the wound by the firſt intention; to remove projecting bones by judicious openings; to reſtrain violent bleeding by long-continued preſſure; to preſerve the limb in a proper and eaſy poſition; and to vary the treatment as ſymptoms may require, have been the means of preſerving many limbs, which were much ſhattered and in a very unpromiſing ſtate.

Although the limb may not admit of a perfect cure, yet it may not prove entirely uſeleſs.

In chronic caſes, the preſent ſtate of the limb, or of the habit of body, may be unfit for the operation, and yet capable of being amended, ſo as afterwards to render it ſafe and proper.

The part where the limb ſhould be taken off may vary according to the extent of the diſeaſe, or mode of operating. Blood veſſels, in the vicinity of the affected part, may be ſo numerous, and ſo much enlarged, as to require the removal of the limb at a greater diſtance than might otherwiſe be choſen.

In all dubious caſes, conſultation is particularly adviſeable, as the moſt experienced and eminent practitioners are not infallible.

The ſubſtitute propoſed by Mr. Parke, of Liverpool, for amputation, in caſes of diſeaſed joints, does him the greateſt credit; much attention and diſcernment are, however, required throughout the cure, when his mode of treatment is adopted. Since the publication of Mr.

Parke's

Parke's work on this ſubject, I have had the ſatisfaction of ſeeing one of his patients in a fair way of recovery after an operation at the knee joint.

I have tried Mr. Moore's machine for diminiſhing pain during operation, and am ſorry to ſay that the pain produced by its application made it highly objectionable; but as no opiate accompanied its uſe, nor many trials were made with it, it would be unfair from my experiments to decide againſt it. It is certain, however, that the application of the tourniquet, for five or ten minutes, gave leſs pain, and ſeemed equally beneficial.—It may deſerve to be conſidered whether ſuch applications may not tend to make the ſtump more liable to future hæmorrhage.

Although healing the wound, after amputation, by the firſt intention, was ſaid to have ſucceeded, yet, as no ſuch practice had been generally adopted, Mr. Alanſon has a juſt claim to the merit of having introduced it, as well as to that of having pointed out an improved method of treating large wounds, particularly thoſe made by operation. From the length of time required to dreſs the flap and ſtump ſeparately, and from the pain which the patients ſuffered,

I had propofed to place them in contact imme-diately after the operation; but the experiment having been difapproved, I declined it until Mr. Alanfon was pleafed to communicate to me verbally his method of amputation, and fubfe-quent treatment. He had not then operated with a flap; but from being fhewn fuch a ftump, he had no doubt of the immediate union fuc-ceeding, and alfo being found a confiderable improvement. In the year 1779 I introduced that, and alfo Mr. Alanfon's method, at our In-firmary, where they have fince been the general practice.

We had been accuftomed, in Mr. Gooch's manner of operating, to referve, by exact mea-furement, a due quantity of the integuments and mufcles, as well as in the ancle a flap of about three or four inches long; but we ufually let the length of the flap depend much on the extent of the difeafe, and, before it was placed in contact with the ftump, a confiderable con-traction was obferved to take place. When the flap and ftump were to be immediately joined, there appeared a greater neceffity for a certain meafurement being previoufly made, that the lips of the wound might meet with exactnefs.

From

From reflecting on what had paffed, I was induced to try the prefervation of a flap, in proportion as the diameter of the limb to its circumference, in the part where the bone was to be fawn through, and, by applying this rule to Mr. Alanfon's method, I found the edges of the wound exactly correfponded.

A little deficiency, or excefs, may eafily be remedied by occafional variations of the retentive applications.

Although as much of the integuments as will cover the mufcular parts be neceffary, yet a due proportion of the latter fhould be attentively referved.

One third of the former will, in general, prove fufficient.

Where the incifion through the mufcles is made ftraight, inftead of floping upwards towards the bone, a fomewhat greater proportion of the integuments may be requifite.

I have referved two thirds of the integuments, and dreffed the ftump as directed by Mr. Mynors, but did not find the cure more fpeedy, nor the ftump fo good. Few inftances will occur where any proportion of the integuments requifite may not more readily be retracted by fetting the ftrictures at liberty with the the point of the amputating knife, than by diſ-ſecting with a ſcalpel.

By depending on the dreſſings he recommends, and the poſition of the patient, for keeping the wound cloſed, I have found the edges more than an inch ſeparated, with ſo ſtrong an adheſion, on the fourth day, that they could not be brought in union; and the cicatrix was broader than when more powerful means of retention are uſed.

The tailed bandage is entitled to preference, whilſt the patient is confined to his bed, and may be made of callico; but the fineſt flannel is better for the circular bandage, from its elaſticity, and its being leſs diſagreeable when wet.

By turning the edge of the knife upwards, in dividing the ſkin, the lips of the wound may be better cloſed, and the cellular membrane prevented from ſtarting out before the ſkin. For this remark I am indebted to Mr. Alanſon.

The ſloping inciſion round the limb, through the muſcles, can only be effected by uſing that edge of the knife near its point. Should there be any difficulty in executing it, it is by no means a reaſon for diſcarding the reſt of the plan; for without it an excellent ſtump may be made, if the reſt of the directions are duly obſerved.

If any doubt ariſes either of the integuments or muſcles having been reſerved in the proportion intended, they may be accurately meaſured before the bone is ſawn through, after each inciſion is made.

Should there be found a deficiency in the former, the ſtrictures muſt be divided to allow a farther retraction; if in the latter, the muſcles immediately round the bone muſt be farther divided, as directed by Mr. Gooch.

In amputations above the knee, I ſeldom uſe a Retractor.

Care ſhould be taken that the bone is not denuded of its perioſteum any higher than the part where it is to be ſawn through.

Inſtead of applying the ſaw horizontally, very great advantage will be found from placing it ſomewhat obliquely on the bone, and ſawing with the hand elevated, as is the cuſtom of carpenters.

We prefer the tenaculum for aſſiſting in ſecuring the arteries.

It has for many years been our conſtant practice to paſs a ſecond ligature (more ſuperficial than than the firſt) upon the femoral artery, and its ſafety is a ſufficient recommendation.

When the ſtump is perfectly cleaned, the ligatures are to be placed over the edge of the wound, not all together, but each neareſt its attachment.

The lips of the wound being properly adapted, three, four, or five ſutures (as repreſented in the plate by croſſes + + +) are to be tied with ſlip knots moderately tight, ſo as to retain the edges together, and the intermediate openings are to be cloſed by ſlips of court plaſter. A circular bandage is to be pinned faſt to the body band, (applied beforehand) and, by gentle turns, carried towards the ſtump, ſo as to ſupport the integuments from retracting, as well as to prevent the other retentives from having too much to hold. If the edges cannot readily be brought to meet, the circular bandage ſhould be applied before the ſutures are tied, and longer as well as broader ſlips of adheſive plaſter may be uſed.

Should the lips of the wound overreach, the means for retention may be looſer: in ſome few caſes the long pledgets may be ſufficient without ſutures or plaſters.

Two b
e
a
2
s
A
f
d
c

Two long pledgets are to be applied acroſs the face of the ſtump, and retained by a tailed bandage, or a few remaining turns of the circular one left for that purpoſe. The patient is to be placed in bed, inclining to the affected ſide, in ſuch a poſture as is eaſy to him, with the limb upon a folded ſheet, and a perſon is to ſit by him to hold the ſtump, particularly during ſleep. A tourniquet ſhould be in conſtant readineſs. Patients of an irritable habit require an opiate, and generally a full doſe, particularly if they have been accuſtomed to ſuch medicines. Attention ſhould be paid to regulate the diet, to prevent coſtiveneſs, and to keep the linen clean.

The time of dreſſing ſhould depend on the diſcharge, or patient's feelings; the fourth or fifth day is as ſoon as is uſually requiſite. The ſutures ſhould ſeldom be removed before the end of a week or ten days; but may (ſhould there be occaſion) be eaſily made looſer. At the firſt dreſſing the wound is ſuperficial, and in general leſs painful and troubleſome than one, managed in the old way, uſually is at the end of two months. All the ligatures are frequently detached in fourteen days. I have known one remain after the ſtump was perfectly cured, but without any bad effect.

When the inflammatory ſymptoms ſubſide, we often uſe ſlips of adheſive plaſter, inſtead of the tow pledgets, to attach the parts more ſtrongly.

In amputation above the knee, the retraction during cure is ſo much greater behind than before, that in Mr. Alanſon's method the cicatrix is not central, but very ſimilar to the ſtump with a flap.

For the laſt three or four years we have preferred amputation with a flap, above as well as below the knee, being of opinion that a thicker covering for the bone is advantageous.

Where amputation by a circular inciſion would either leave too ſhort a ſtump, or fail to remove the whole of the diſeaſe, as in the caſe of ulcers above the knee, it frequently happens that a flap may be formed out of the ſound parts, and a longer ſtump be obtained.

The anterior part of the limb above the knee is moſt ſuitable; but the hinder part will alſo admit of a flap being made, ſhould diſeaſe render it neceſſary.

The flap has been made with the catlin, as in the ancle, which has been eſteemed preferable able to detaching it with a ſcalpel, as is practiſed by ſome; but the common catlin is too ſhort for a flap above the knee. One of ſix or eight inches long, and rather broader than the catlin in common uſe, is required.

Where the flap is preſerved anteriorly, the inſtrument is to be carried as cloſe as poſſible to the os femoris; but if behind, no deeper than is ſufficient to make the flap of a proper thickneſs; and the limb, in this caſe, ſhould be turned, as is directed in the operation at the ancle.

When a flap is neceſſary above the left knee, as repreſented in the plate, the circumference of the thigh is to be taken at the part where the bone is to be ſawn through, as at *a*, *e*, *b*, and the length of the flap from its baſis *e*, to its apex *f*, ſhould be about a third of the circumference; the breadth of the flap ſhould be nearly equal to its length, and ſomewhat broader at the baſis than at the apex, as marked by the lateral lines *a*, *e*, *b*, *d*. If the circumference at *a*, *e*, *b*, is twelve inches, the flap A ſhould be nearly four inches ſquare; but where the circumference is greater, and the ſtump would be too ſhort, if a flap of a proportionate length were preſerved, a portion of the hinder

 parts parts may be ſaved, ſo as to meet the apex of the flap when the wound is dreſſed; this is to be effected by the circular inciſion being made in the line *s*, through the integuments, and that through the muſcles at No. 2, which, at other times, would be about the proper mark for the firſt inciſion.

Should the diſeaſe in the limb extend far above the knee, and equally in every part, the circular inciſion would then have the advantage of the flap operation, for the ſame reaſon of being capable of removing the whole of the diſeaſe, and obtaining the longeſt ſtump.

The meaſurements may be taken with a ſilk yard band, marked with inches, and the lines preſerved, by a pencil of red chalk, or ſome ſuch colouring ſubſtance. This may be done whilſt the patient is in bed. The parts for inciſion, when marked, are an eaſier guide for the operation than either tape or adheſive plaſter.

A ſurgeon ſhould have in conſtant readineſs a regular liſt of every thing that can be requiſite for each capital operation, but eſpecially for ſuch as may be required ſuddenly. However trivial ſuch exactneſs may appear to ſome, it will be found highly beneficial. An alphabetical liſt of the different writers on the ſubject, to which he may occasionally refer, will likewise be very useful.

For lithotomy, a table of about a yard high is requisite; but one somewhat lower is more convenient for amputation. Mr. Smith, surgeon to the Bristol Infirmary, has invented a table capable of being occasionally elevated.

The compression of the tourniquet is best made by having a bolster applied on the course of the femoral artery, (as high as the bone will admit) and secured by tapes long enough to tie, or fastened by buckles. The bolster may be about four inches long, and two broad. Two tourniquets should always be provided, and the buckles and screws be well guarded by strong leather, stuffed with wool or hair.

If the blade of the catlin be eight inches long, the circular incisions may be made with it; but if the limb be thick, the amputating knife is preferable.

The retractor may be made of linen, or as directed by Mr. Gooch.

The saw used at our hospital is made by Mr. Squires, saw maker, in Broad Street, Carnaby Market, London; its blade, though not more than nine inches long, and two broad, is sufficiently strong, and works remarkably easy.

The

The incifive forceps, for removing any irregularities in the bone after it is fawn through, fhould be very ftrong.

The ligatures fhould not be too much waxed, and fhould be of fufficient thicknefs.

The futures fhould be armed upon ftraight needles of about two inches and a half long, which pafs much eafier than curved ones.

The bandage, to be faftened round the patient's body, fhould be about three or four fingers broad, and of linen or callico. The circular bandage fhould be fix yards long, and near three inches broad, and made of the fineft flannel. A fquare of about fixteen or feventeen inches of callico or flannel is fufficient to make the tailed bandage.

Where the flips of adhefive plafter are within the compafs of its fize, court plafter deferves the preference, on account of its fticking when wet. The tow pledget fhould be about fifteen inches long, made pointed, and about four inches broad in the center. The patient, operator, and affiftants, fhould be fuitably dreffed; the apparatus fo regularly difpofed as to be reached by filent, or fome figns unintelligible to the patient, whofe eyes fhould be covered; and and every attention ſhould be paid not to occa-ſion unneceſſary diſtreſs.

The parts being marked, the bolſter and tourniquet ſufficiently tight, and under the management of an aſſiſtant, and the patient placed to the ſatisfaction of the operator, the latter is to ſtand on the inſide, if it is the left limb, and *vice verſa*, and to elevate as much as he can the flap A (ſee the plate) with his left hand, whilſt he paſſes the catlin, in an horizontal direction, with its upper edge in a line with *b*, *e*, *a*, and as deep as the os femoris will admit, carrying the inſtrument with a ſmart ſtroke downwards towards the knee, and bringing it out at *d*, *c*.

The flap, thus formed, being reverted by the aſſiſtant, who holds the ſkin tight, a circular inciſion is to be made through the remaining undivided integuments by one ſtroke, beginning at No. 2.

As ſoon as the ſtrictures are divided to allow ſufficient retraction of the integuments, a circular inciſion through the muſcles, obliquely upwards, is to be made as deep as the bone, and correſponding with the line of circumference or baſis of the flap *a*, *e*, *b*.

The os femoris being circularly denuded to a proper height, the bone is to be ſawn through in in the manner already advifed; in effecting which the parts may generally be held back by an affiftant; if not, a retractor is to be ufed, and attention fhould be paid that the limb is held low enough to allow the operator to faw with the handle of the faw elevated. If any roughnefs is left on the end of the bone, or any irregular ends of tendon, they fhould be carefully removed; the arteries fecured as expeditioufly as poffible; the ftump well cleaned; the ligatures each placed feparately over the edge; and the lips of the wound retained in contact by futures and adhefive plafters, as well as the whole properly fupported by bandage, as has been already mentioned.

To be able to adapt the lips of the wound exactly by previous meafurement, and retain them in fuch contact as that, in a few days, there fhall remain only a fuperficial wound; to leffen the pain and fymptomatic fever; to prevent hæmorrhage and copious difcharge; to heal the ftump much more fpeedily, as well as to form one much more able to refift any injury, and be more ufeful, are advantages peculiarly obvious in weak, irritable, and fcrophulous habits, as well as highly deferving the attention tention of practitioners in camps and hot climates.

These methods have been found to succeed, in a variety of unpromising cases, in patients of all ages, and have been practised with peculiar advantage where the patient has been extremely reduced, or the limb much diseased. Even where the integuments and muscles have been found much diseased, and where the bone has been soft enough to be cut through by the knife, neither any copious discharge nor any exfoliation has delayed the cure.

Success has attended this mode of treatment in patients who were far advanced in years, and who were so reduced as to faint upon motion from one place to another. In these cases the preservation of the patient's life appeared to depend on the advantages derived from this plan.

No patient has appeared to suffer from tediousness in the operation; if any additional time is required, it is chiefly employed in adjusting and retaining the lips of the wound together: no inflammation from the retentive means has happened to prove them in any degree objectionable; no trouble from matter forming, or the parts failing to unite; nor has there been any sensible exfoliation to obstruct the cure.

A few cafes have occurred where a fuperficial ulcer has remained a week or two beyond the month. An inftance or two has happened of hæmorrhage after the parts appeared firmly united, and more than a week after the operation. This circumftance happened to a patient of mine, fo as to oblige me to divide the parts until the bleeding artery was denuded. When the veffel was fecured, the futures, which had not been withdrawn, again retained the edges, and the wound was ftill healed in five weeks.

The pain of dividing the parts did not appear to exceed what I have obferved in feparating the dreffings of an open ftump for the fame purpofe; and the union of the wound had acted as a comprefs, and prevented the bleeding from being fo rapid.

The integuments had formed a firmer attachment than the mufcular parts. In fome parts there appeared an inorganic mafs, feparable by the fingers, but with fibres fhooting through it, which required divifion with the fcalpel, and which were evidently fenfible.

I have been informed of an inftance of the bone being found, at the firft dreffing, protruded through the fkin; but in this cafe an undue undue proportion of the integuments had been preſerved.

At an hoſpital I viſited, I ſaw a caſe, in which amputation, after Mr. Alanſon's method, was ſaid to have been performed two months before, and where the ſtump was more open than under the old management. In this caſe, although all the ligatures were out, and the granulations perfectly clear, no circular bandage was applied, and the dreſſings were lint, fomentations, and cataplaſm. The perioſteum had receded above half an inch from the end of the bone, and the patient complained much when dreſſed. Some have applied lint at the firſt dreſſing, and others have neglected to continue the means for cloſing the wound, and from thence have condemned the method.

Theſe failures will be found to have depended more on deviations from, than on defects in, the method of operating, or ſubſequent treatment.

When the plan is duly attended to, it will afford the greateſt ſatisfaction to the ſurgeon, and the greateſt eaſe to the patient. The progreſs of the cure cannot fail to give the greateſt pleaſure; and the degree of pain, if any other complaint attends, is generally deſcribed as being leſs in the ſtump. A patient, with a compound fracture in both legs, regretted much that both had not been amputated, from the additional pain that was given her by the fractured leg, whilſt the ſtump, ſhe obſerved, ſeldom called her attention.

A patient of mine, very lately, obſerved, that an eryſipelas, which attacked his arm the day after an amputation of his leg, was much greater diſturbance to him than his ſtump.

Although it is true that a firmer adheſion takes place in the integuments than the muſcular parts, in the ſame ſpace of time; yet, unleſs the whole cure could be performed in leſs time, or the mode of operating was in any reſpect preferable, I ſee no reaſon for objecting to ſaving a due proportion of muſcular parts.

I amputated the limbs of two patients, above the knee, within a day or two. In one of thoſe caſes, where both the habit of body, and ſtate of the limb, ſeemed moſt promiſing, I followed Mr. Mynors's plan; in the other, I performed the operation with the flap. Both the cures were as near as poſſible equally ſpeedy; but, in the former, the cloſing of the wound was not ſo quick or perfect, and the cicatrix was broader, and more central than the other.

If

If the end of the ſtump wears thinner, (as in many caſes it evidently does) it muſt be beneficial to have a thicker cuſhion preſerved.

When a thick muſcular cuſhion is ſaved, I have no doubt of many patients being able to bear preſſure, on the end of the ſtump, which is as ſmooth as the ſkin upon the back of the hand; but if preſſure at the end was not tolerable, ſuch a ſtump appears to be much better fitted to reſiſt any injury, wherever the preſſure is thought proper to be made. I lately met the young woman whoſe limb I amputated above the knee, by Mr. Alanſon's verbal directions, in 1779, and aſked her what part of her ſtump touched the artificial boot when ſhe walked; ſhe replied, both the end and the ſides, and that ſhe had never had any thing ailed the ſtump ſince ſhe left the hoſpital. Some time ago I directed the man, who makes artificial legs for our Infirmary, to be careful that thoſe made for my patients ſhould not only preſs on the ſides, but alſo on the end of the ſtump, and I have not yet heard of any inconvenience having enſued.

The method of approximating the flap in the ancle, is already fully deſcribed, by Mr. Jones, in the ninth volume of the Edinburgh Medical

Com-

Commentaries. My experience of its utility enables me to add my teſtimony in its favour.

Although many of the preceding remarks may have already appeared in print, yet their having been practically proved, in a great number of caſes, may tend to eſtabliſh more generally the improvements I have been deſcribing.

Succeſs in the ſpeedy union of wounds after amputation, ſoon induced us to try the ſame method after other capital operations, and alſo in the cure of large wounds; fewer ligatures, and the dimenſions of the wound being generally leſs, ſoon convinced us that the plan was frequently more manageable, and equally ſucceſsful.

I have at preſent a patient, whoſe arm, from above the elbow to the fingers, was torn by a ſcribbling mill, and a conſiderable portion of the integuments entirely removed. In this caſe, by ſutures, and adheſive plaſters, the ſize of the wound has been much diminiſhed, although its edges could not be brought in contact.

## Figures and Tables

**Figure f1:**